# Supplementary material for: Concerted IL-25R and IL-4Rα signaling drive innate type 2 effector immunity for optimal helminth expulsion
Source: eLife. 2018 Sep 21;7:e38269. doi: 10.7554/eLife.38269 (PMC6173579; doi:10.7554/eLife.38269)
Supplement: Transparent reporting form [file elife-38269-transrepform.docx]

***eLife’s* transparent reporting form**

We encourage authors to provide detailed information *within their submission* to facilitate the interpretation and replication of experiments. Authors can upload supporting documentation to indicate the use of appropriate reporting guidelines for health-related research (see [EQUATOR Network](http://www.equator-network.org/%20)), life science research (see the [BioSharing Information Resource](https://biosharing.org/)), or the [ARRIVE guidelines](http://www.plosbiology.org/article/info:doi/10.1371/journal.pbio.1000412) for reporting work involving animal research. Where applicable, authors should refer to any relevant reporting standards documents in this form.

If you have any questions, please consult our Journal Policies and/or contact us: [editorial@elifesciences.org](mailto:editorial@elifesciences.org).

**Sample-size estimation**

- You should state whether an appropriate sample size was computed when the study was being designed
- You should state the statistical method of sample size computation and any required assumptions
- If no explicit power analysis was used, you should describe how you decided what sample (replicate) size (number) to use

Please outline where this information can be found within the submission (e.g., sections or figure legends), or explain why this information doesn’t apply to your submission:

Sample sizes were set at n=5 for preliminary experiments and all experiments repeated at least once, hence n≥10 wherever availability of appropriate transgenic genotypes was not limiting. This size is based on the statistical consideration that to achieve a probability of <0.05 with a normal distribution and a standard deviation of 5% of the mean, a minimum group size of 4 is necessary. Because individual mice may need to be excluded or

culled, a group size of 5 is needed to ensure valid results from each experiment.

**Replicates**

- You should report how often each experiment was performed
- You should include a definition of biological versus technical replication
- The data obtained should be provided and sufficient information should be provided to indicate the number of independent biological and/or technical replicates
- If you encountered any outliers, you should describe how these were handled
- Criteria for exclusion/inclusion of data should be clearly stated
- High-throughput sequence data should be uploaded before submission, with a private link for reviewers provided (these are available from both GEO and ArrayExpress)

Please outline where this information can be found within the submission (e.g., sections or figure legends), or explain why this information doesn’t apply to your submission:

Figure Legends state number of biological replicate experiments performed, in all cases ≥2, and stating whether replicates were independent biological replicates or technical replicates. Data from individual mice are presented for in vivo experiments; no individuals were excluded. No sequence data are presented.

**Statistical reporting**

- Statistical analysis methods should be described and justified
- Raw data should be presented in figures whenever informative to do so (typically when N per group is less than 10)
- For each experiment, you should identify the statistical tests used, exact values of N, definitions of center, methods of multiple test correction, and dispersion and precision measures (e.g., mean, median, SD, SEM, confidence intervals; and, for the major substantive results, a measure of effect size (e.g., Pearson's r, Cohen's d)
- Report exact p-values wherever possible alongside the summary statistics and 95% confidence intervals. These should be reported for all key questions and not only when the p-value is less than 0.05.

Please outline where this information can be found within the submission (e.g., sections or figure legends), or explain why this information doesn’t apply to your submission:

Statistical methods are described in the Methods section, and in addition specified as appropriate in each Figure Legend; raw data (individual mouse data) are presented for in vivo experiments, and representative raw FACS plot data also presented; number of replicates, values of N and form of mean/SEM stated. Significance is indicated on all graphs in bands, <0.05, <0.01 and <0.001; because sample sizes are relatively small, the exact p-values overly depend on variance rather than effect size, hence banding avoids false accuracy of p-values.

(For large datasets, or papers with a very large number of statistical tests, you may upload a single table file with tests, Ns, etc., with reference to sections in the manuscript.)

**Group allocation**

- Indicate how samples were allocated into experimental groups (in the case of clinical studies, please specify allocation to treatment method); if randomization was used, please also state if restricted randomization was applied
- Indicate if masking was used during group allocation, data collection and/or data analysis

Please outline where this information can be found within the submission (e.g., sections or figure legends), or explain why this information doesn’t apply to your submission:

In vivo experiments involved laboratory mice bred for the purpose and all mice available were randomly allocated into the different groups for each experiment. All assays were objective (eg flow cytometry, ELISA) and hence no masking was applied.

**Additional data files (“source data”)**

- We encourage you to upload relevant additional data files, such as numerical data that are represented as a graph in a figure, or as a summary table
- Where provided, these should be in the most useful format, and they can be uploaded as “Source data” files linked to a main figure or table
- Include model definition files including the full list of parameters used
- Include code used for data analysis (e.g., R, MatLab)
- Avoid stating that data files are “available upon request”

Please indicate the figures or tables for which source data files have been provided:

All data are contained in the main manuscript and figures, no additional data files are required.
